# Complete Genome Sequence of *Bifidobacterium longum* W11 (LMG P-21586), Used as a Probiotic Strain

**DOI:** 10.1128/genomeA.01659-16

**Published:** 2017-03-09

**Authors:** Rosanna Inturri, Marco Ventura, Patricia Ruas-Madiedo, Gabriele Andrea Lugli, Giovanna Blandino

**Affiliations:** a Department of Biomedical and Biotechnological Sciences, University of Catania, Catania, Italy; b Laboratory of Probiogenomics, Department of Life Sciences, University of Parma, Parma, Italy; c Department of Microbiology and Biochemistry of Dairy Products, Instituto de Productos Lácteos de Asturias, Consejo Superior de Investigaciones Científicas (IPLA-CSIC), Villaviciosa, Asturias, Spain

## Abstract

We report the complete genome sequence of *Bifidobacterium longum* W11 (LMG P-21586) isolated from the intestinal microbiota of a healthy man. The analysis of the sequence may provide insights into the microbiological characteristics and the functional activity of this probiotic strain.

## GENOME ANNOUNCEMENT

Members of the genus *Bifidobacterium* are nonmotile non-spore-forming Gram-positive polymorphic anaerobic rods, with a high G+C DNA content. *Bifidobacterium* spp. ferment carbohydrates to produce acetic acid and lactic acid but no carbon dioxide, and they metabolize glucose via the fructose-6-phosphate phosphoketolase shunt (1–3). This genus represents an important commensal group of the human intestinal microbiota, particularly during breastfeeding (4). *Bifidobacterium longum* subsp. *longum* W11 (LMG P-21586) has been isolated from the intestinal microbiota of a healthy man and has been studied for some microbial characteristics (5–8).

Genomic DNA of *B. longum* W11 was subject to whole-genome sequencing using MiSeq (Illumina, United Kingdom) at GenProbio s.r.l. (Parma, Italy). Paired-end reads obtained from targeted genome sequencing were used as input for the genome assemblies through the MEGAnnotator pipeline (9). The MIRA program (version 4.0) was used for the *de novo* assembly, while proteins encoded by open reading frames (ORFs) were predicted using Prodigal. The circular chromosome contains 2,329,981 bp with 1,884 ORFs, with these total numbers being higher than those in the representative *B. longum* NCC2705. The average of G+C content of the genome is 59.9%.

The complete genome sequence of *B. longum* W11 may be useful to better understand the health-promoting characteristics of this probiotic strain and to clarify the mechanisms involved in its interaction with the host.

### Accession number(s).

This whole-genome shotgun project has been deposited at DDBJ/EMBL/GenBank under the accession number MRBG00000000. The version described in this paper is version MRBG01000000.
